# ^1^H, ^15^N and ^13^C resonance assignments of the HR1c domain of PRK1, a protein kinase C-related kinase

**DOI:** 10.1007/s12104-020-09954-7

**Published:** 2020-06-04

**Authors:** Georgios Sophocleous, George Wood, Darerca Owen, Helen R. Mott

**Affiliations:** 1 Department of Biochemistry, 80, Tennis Court Road, Cambridge, CB2 1GA UK; 2 Present Address: Department of Pathology, 10, Tennis Court Road, Cambridge, CB2 1QP UK

**Keywords:** Small GTPase, Protein Kinase C related kinase, PKN, HR1 domain, Coiled-coil

## Abstract

PRK1 is a member of the protein kinase C-related kinase (PRK) family of serine/threonine kinases and a downstream effector of Rho GTPases. PRK1 has three N-terminal Homology Region 1 (HR1) domains (HR1a, HR1b and HR1c), which form antiparallel coiled coils that interact with Rho family GTPases. PRK1 also has a C2-like domain that targets it to the plasma membrane and a kinase domain, which is a member of the protein kinase C superfamily. PRK1 is involved in cytoskeletal regulation, cell adhesion, cell cycle progression and the immune response, and is implicated in cancer. There is currently no structural information for the HR1c domain. The ^1^H, ^15^N and ^13^C NMR backbone and sidechain resonance assignment of the HR1c domain presented here forms the basis for this domain’s structural characterisation. This work will also enable studies of interactions between the three HR1 domains in an effort to obtain structural insight into the regulation of PRK1 activity.

**Electronic supplementary material:**

The online version of this article (doi:10.1007/s12104-020-09954-7) contains supplementary material, which is available to authorized users.

## Biological context

Protein kinase C-related kinase 1 (PRK1) is a member of the PRK family of serine/threonine kinases, which is within the AGC group of kinases. The PRK proteins were first identified as effector kinases of the small G protein RhoA (Watanabe et al. 1996; Amano et al. 1996). Several roles of PRK1 in cytoskeletal regulation have been described, including phosphorylation of vimentin (Matsuzawa et al. 1997), neurofilament assembly disruption (Manser et al. 2008), interaction with the actin cross-linking protein α-actinin (Mukai et al. 1997) and insulin-induced stress fibre disruption (Dong et al. 2000). PRK1 has also been linked to androgen receptor signalling and transcriptional regulation (Metzger et al. 2003, 2008), the immune response (Park et al. 2016), cell cycle progression (Schmidt et al. 2007), thromboxane signalling (O’Sullivan et al. 2015) and mTOR signalling (Yang et al. 2017; Wallroth et al. 2019). PRK1 is overexpressed in human prostate cancer (Metzger et al. 2003; O’Sullivan et al. 2017) and has also been implicated in ovarian cancer (Galgano et al. 2009) and in the migration of bladder tumour cells (Lachmann et al. 2011).

PRK1 has three N-terminal HR1 domains, HR1a, HR1b and HR1c. These are antiparallel coiled coils of approximately 10 kDa that interact with the nucleotide-sensitive switch regions of Rho GTPases as shown in the structures of RhoA with HR1a (Maesaki et al. 1999) and Rac1 with HR1b (Modha et al. 2008). PRK1 also has a C2-like domain, which targets the protein to the plasma membrane (reviewed in Corbalán-García and Gómez-Fernández 2010) and a C-terminal catalytic domain, which belongs to the protein kinase C family (reviewed in Pearce et al. 2010; Arencibia et al. 2013; Chamberlain et al. 2014). The three HR1 domains together with the C2 domain comprise the regulatory region of the PRKs.

PRK1 is activated by phosphoinositide-dependent protein kinase 1 (PDK1) phosphorylation of its activation loop (Dong et al. 2000) and by lipids such as PIP_2_, PIP_3_ and arachidonic acid (Palmer et al. 1995; Mukai et al. 1994; Morrice et al. 1994; Kitagawa et al. 1995; Peng et al. 1996; Yu et al. 1997; Mukai and Ono 2006; Falk et al. 2014). Small GTPases like RhoA and Rac1 can also activate PRK1 activity (Amano et al. 1996; Lu and Settleman 1999) and it is thought that RhoA binds to HR1a to relieve autoinhibition mediated by a pseudosubstrate region in this domain (Kitagawa et al. 1996).

The HR1a and HR1b domains both bind to Rho proteins and their structures have been characterised. There is currently no structural information on the HR1c domain and it is unknown whether this domain is structured and what role it plays in PRK1 regulation. There is also no information on the entire HR1 region and particularly on whether the three HR1 domains interact with each other or exist independently in solution. Here we present the ^1^H, ^15^N and ^13^C NMR resonance assignment of the PRK1 HR1c domain. This will form the basis for structure determination and for investigating interactions between the three PRK1 HR1 domains, providing much needed structural insight into PRK1 regulation.

## Methods and experiments 

### Protein expression and purification

Human PRK1 HR1c (residues 201–297) was expressed in pGEXHISP (Hutchinson et al. 2011) with a stop codon engineered to prevent the translation of the C-terminal His_6_ tag. The HR1c domain was expressed as a GST fusion in *E.coli* BL21 (DE3) Rosetta2 pLysS. Overnight cultures were diluted 1 in 10, grown at 37 ^o^C to an A_600_ of 0.8 and induced by adding 0.1 mM isopropyl β-d-1-thiogalactopyranoside. The cultures were then incubated at 20 ^o^C for 20 h. For NMR experiments, isotopically labelled protein was produced by growing *E.coli* in M9 media supplemented with 1 g/L ^15^NH_4_Cl (Sigma-Aldrich) and 3 g/L ^13^C-glucose (Cambridge Isotope Laboratories). The protein was purified using glutathione agarose beads, eluted by adding HRV 3C protease to cleave the N-terminal GST-tag, further purified by size exclusion chromatography on a Superdex75 16/60 column (GE Healthcare) and concentrated in an Amicon Ultra-4 Centrifugal Filter Unit (Millipore). Protein concentration was determined by amino acid analysis (Protein and Nucleic Acid Chemistry Facility, Department of Biochemistry, University of Cambridge).

### NMR spectroscopy

NMR spectra were recorded at 298 K with 1.6 mM ^15^N-labelled HR1c or 1.2 mM ^15^N, ^13^C-labelled HR1c in 20 mM sodium phosphate pH 7.3, 150 mM NaCl, 0.05% NaN_3_, 10% D_2_O. ^15^N-HSQC, ^15^N-separated NOESY (150 ms mixing time), ^15^N-separated TOCSY (60 ms mixing time), HNCA, HNCACB, HN(CO)CA, HN(CO)CACB, ^13^C-HSQC and ^13^C-separated HCCH-TOCSY experiments were recorded on a Bruker DRX500. A ^13^C-separated NOESY experiment (100 ms mixing time) was recorded on a Bruker AV800. NMR data were processed using Azara (Wayne Boucher, University of Cambridge).

Backbone and side-chain resonance assignment was carried out in CCPN Analysis v2.3 (Vranken et al. 2005). Standard methodology (Gardner and Kay 1998) was used to carry out the backbone assignment using the ^15^N-separated NOESY, ^15^N-separated TOCSY, HNCA, HNCACB, HN(CO)CA, HN(CO)CACB experiments with reference to the ^15^N-HSQC experiment. The ^13^C-separated HCCH-TOCSY and ^13^C-separated NOESY experiments were used with reference to the ^13^C-HSQC, in addition to the above experiments, for the assignment of side-chain resonances. Side-chain amides were assigned using the ^15^N-separated NOESY and the ^13^C-separated NOESY. Aromatic side-chains were assigned using the ^15^N-separated NOESY and the ^13^C-separated NOESY spectra with reference to the aromatic region of the ^13^C-HSQC spectrum.

## Extent of assignment and data deposition

**Fig. 1:**
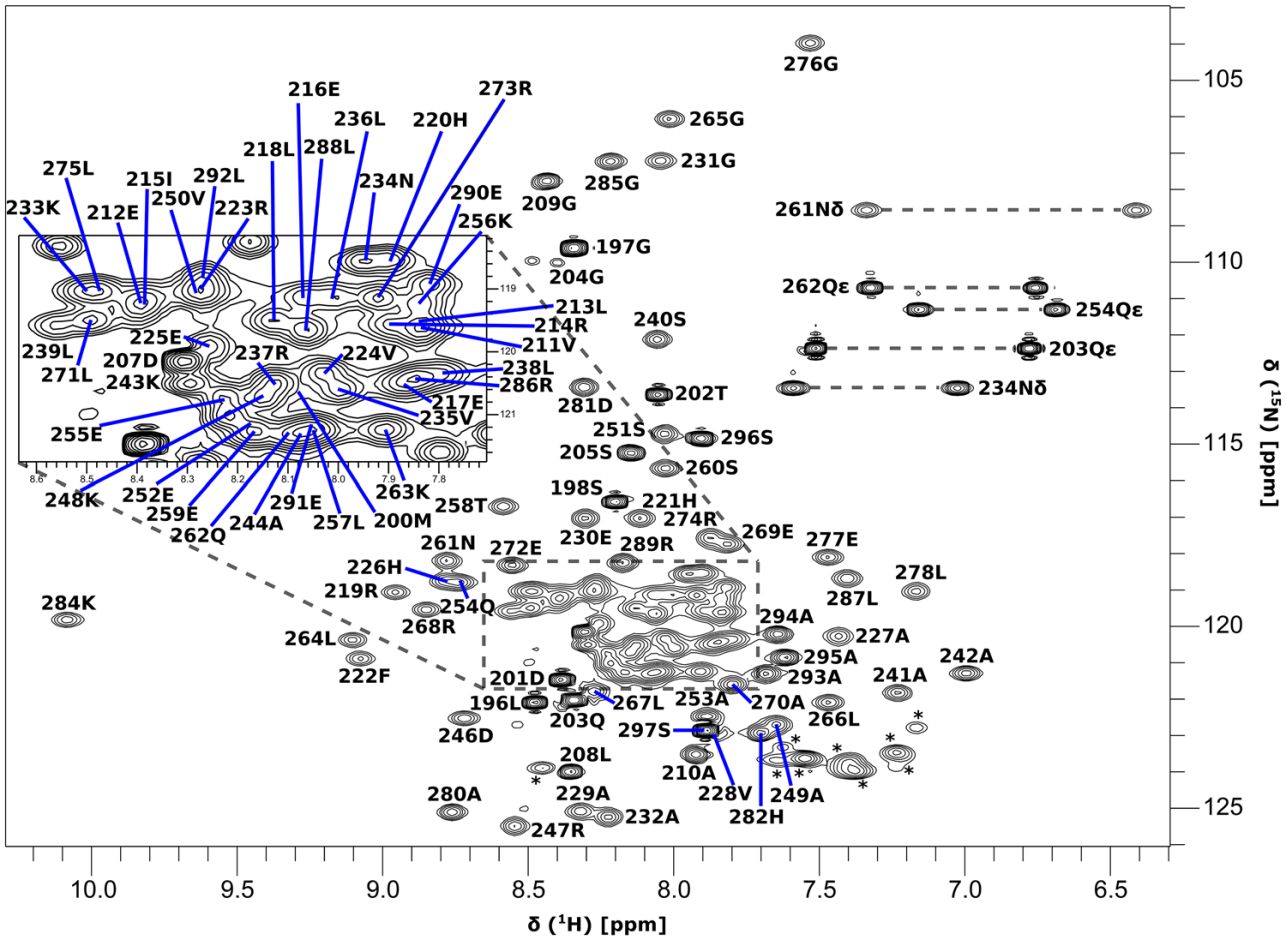
^15^N-HSQC recorded on 1.6 mM ^15^N-labelled PRK1 HR1c in 20 mM sodium phosphate pH 7.3, 150 mM NaCl and 10% D_2_O on a Bruker DRX500 at 298 K. The assignments are indicated for all assigned resonances. The arginine side-chain H_ε_N_ε_ resonances are aliased in the ^15^N dimension and are indicated by an asterisk

The PRK1 HR1c domain gave well-dispersed spectra as shown by the quality of the ^15^N HSQC spectrum (δ^Η^ = 6.4–10.1 ppm and δ^N^ = 104–126 ppm) (Fig. 1). The construct used includes PRK1 HR1c residues 201–297 and an additional 7 N-terminal residues (GPLGSHM) encoded by the expression vector, and the majority of resonances were assigned (Table 1). All the backbone NH resonances were assigned, with the exception of the His in the N-terminal linker. All Asn and Gln NH_2_ resonances were assigned. Nine out of the 10 expected Arg H_ε_N_ε_ peaks were observed in the HSQC (Fig. 1) but could not be unambiguously assigned, although one Arg H_ε_ was assigned in the ^13^C-separated NOESY. All other sidechain resonances were assigned except those that rapidly exchange with the solvent: OH groups, primary amines (N-terminal and Lys sidechains), Arg H_η_/N_η_, and His H_δ1_/N_δ1_ and H_ε2_/N_ε2_.

**Table 1:** Extent of assignments of PRK1 HR1c

	Atom	Number of expected	Number assigned	Percentage assignment
PRK1 HR1c	HN^a^	98	97	99.0%
	^15^NH^a^	104	97	93.3%
	^13^Cα	104	104	100.0%
	^1^Hα	104	104	100.0%

^a^Backbone only

**Fig. 2:**
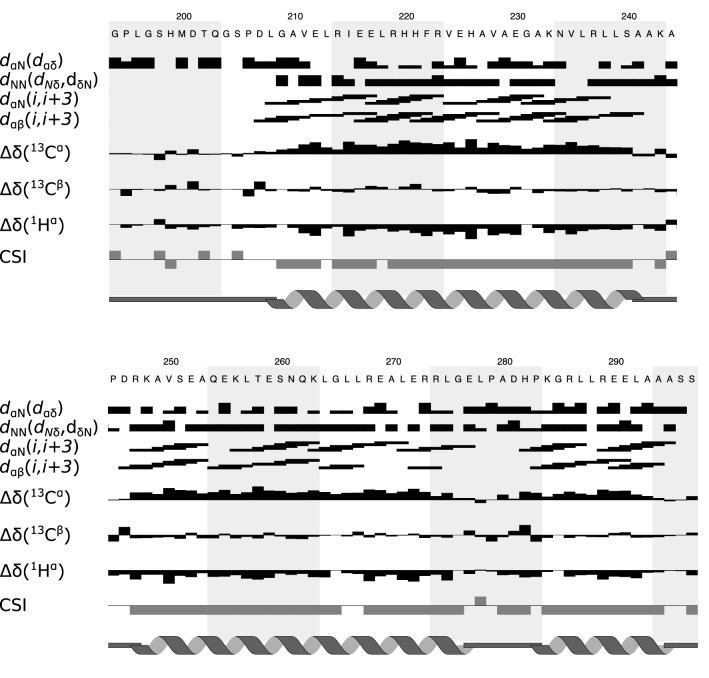
Summary of secondary structure of the PRK1 HR1c domain. The plot summarises the sequential and medium-range NOEs observed in ^15^N-separated and ^13^C-separated NOESY experiments. The height of the bars represents the strength of the following NOEs: (1) d_αN_ (d_αδ_)—NOEs between H_α_ of residue i and NH of residue i + 1 or H_δ_ of an i + 1 Proline; (2) d_NN_ (d_Nδ_)—NH of residue i and NH of residue i + 1 or H_δ_ of an i + 1 Proline. The next 2 rows show the short-range, i to i + 3 NOEs observed in α-helical regions. Δδ represents the chemical shift deviation from random coil for ^13^C_α_, ^13^C_β_ and ^1^H_α_ where the size of the bar represents the size of the shift deviation. The overall score for the secondary shifts, the chemical shift index (CSI), is represented as grey boxes. A value of + 1 denotes an extended, β-sheet-like structure and a value of −1 an α-helical structure. Secondary structure was predicted from the chemical shifts using TALOS-N and is shown with a cartoon representation

A Chemical Shift Index (CSI) of + 1 or −1 was generated from the difference in backbone chemical shifts (C_α_, C_β_ and H_α_) from the random coil positions (Wishart and Sykes 1994). Figure 2 shows the short-range NOEs and the CSI for each residue. The backbone chemical shifts were also submitted to TALOS-N (Shen and Bax 2013) to predict the protein’s secondary structure, which is also shown in Fig. 2. Taken together the CSI, TALOS-N and short-range NOEs allow the secondary structure to be determined. The data suggest that the PRK1 HR1c domain comprises three α-helices that are connected by short, unstructured regions. The first two helices are likely to form an anti-parallel coiled coil akin to other HR1 domains whose structures are known: the PRK1 HR1a (Maesaki et al. 1999), PRK1 HR1b (Owen et al. 2003), TOCA1 HR1 (Watson et al. 2016) and CIP4 HR1 (Kobashigawa et al. 2009) domains. Chemical shift assignment of the PRK1 HR1c domain will enable the structure determination using distance and torsion angle restraints determined from NMR experiments. The full backbone assignment will also allow investigation into inter-domain interactions between the three HR1 domains in PRK1. This will provide a structural understanding of how PRK1 activity may be regulated. The chemical shifts have been deposited in the BMRB, accession number 50216.

## Electronic supplementary material

Below is the link to the electronic supplementary material.

Electronic supplementary material 1 (PNG 1356 kb)
